# Dr. John B. L’Hommedieu

**Published:** 1913-03

**Authors:** 


### OBITUARIES.

Dr. John B. L'Hommedieu, N. Y. University, 1890, of Rosebank, Borough of Richmond, died suddenly February 21, aged 47. He was born in Medina.

